# Molecular Assays for Determining *Mycobacterium leprae* Viability in Tissues of Experimentally Infected Mice

**DOI:** 10.1371/journal.pntd.0002404

**Published:** 2013-08-22

**Authors:** Grace L. Davis, Nashone A. Ray, Ramanuj Lahiri, Thomas P. Gillis, James L. Krahenbuhl, Diana L. Williams, Linda B. Adams

**Affiliations:** 1 School of Veterinary Medicine, Louisiana State University, Baton Rouge, Louisiana, United States of America; 2 Department of Health and Human Services, Health Resources and Services Administration, Healthcare Systems Bureau, National Hansen's Disease Programs, Baton Rouge, Louisiana, United States of America; Fondation Raoul Follereau, France

## Abstract

**Background:**

The inability of *Mycobacterium leprae* to grow on axenic media has necessitated specialized techniques in order to determine viability of this organism. The purpose of this study was to develop a simple and sensitive molecular assay for determining *M. leprae* viability directly from infected tissues.

**Methodology/Principle Findings:**

Two *M. leprae*-specific quantitative reverse transcription PCR (qRT-PCR) assays based on the expression levels of *esxA*, encoding the ESAT-6 protein, and *hsp18*, encoding the heat shock 18 kDa protein, were developed and tested using infected footpad (FP) tissues of both immunocompetent and immunocompromised (athymic *nu/nu*) mice. In addition, the ability of these assays to detect the effects of anti-leprosy drug treatment on *M. leprae* viability was determined using rifampin and rifapentine, each at 10 mg/kg for 1, 5, or 20 daily doses, in the athymic *nu/nu* FP model. Molecular enumeration (RLEP PCR) and viability determinations (qRT-PCR) were performed via Taqman methodology on DNA and RNA, respectively, purified from ethanol-fixed FP tissue and compared with conventional enumeration (microscopic counting of acid fast bacilli) and viability assays (radiorespirometry, viability staining) which utilized bacilli freshly harvested from the contralateral FP. Both molecular and conventional assays demonstrated growth and high viability of *M. leprae* in *nu/nu* FPs over a 4 month infection period. In contrast, viability was markedly decreased by 8 weeks in immunocompetent mice. Rifapentine significantly reduced bacterial viability after 5 treatments, whereas rifampin required up to 20 treatments for the same efficacy. Neither drug was effective after a single treatment. In addition, host gene expression was monitored with the same RNA preparations.

**Conclusions:**

*hsp18* and *esxA* qRT-PCR are sensitive molecular indicators, reliably detecting viability of *M. leprae* in tissues without the need for bacterial isolation or immediate processing, making these assays applicable for in vivo drug screening and promising for clinical and field applications.

## Author Summary

*M. leprae*, the causative agent of leprosy, cannot be grown on laboratory culture media. This characteristic, along with its extremely long generation time of 12–14 days, makes the study of the pathogenicity of this organism and the experimental testing of new drugs for the treatment of leprosy extremely difficult. We developed two *M. leprae*-specific quantitative reverse transcription PCR assays and tested their utility as biological markers of *M. leprae* viability in tissue specimens. These assays could detect high viability of bacilli growing in immunosuppressed mice as well as the inhibitory effects of anti-leprosy drug treatment, or of the host immune system in immunocompetent mice. The RNA preparations were also successfully used for detection of host gene expression. The application of these assays to various experimental models would benefit characterization of the infection or novel drug screening. Furthermore, because these assays utilize fixed tissues, their potential application to clinical and field settings could enable monitoring of *M. leprae* viability in conjunction with the host immune response during treatment.

## Introduction

*Mycobacterium leprae*, an obligate intracellular pathogen and the etiologic agent of leprosy, cannot be grown in axenic medium. This characteristic, in conjunction with its extremely slow generation time of 12–14 days, hinders experimentation addressing even the most fundamental questions regarding its genetics, metabolism, sensitivity to anti-microbials, and pathogenicity. Live animal models are required for bacterial cultivation. Limited growth occurs in the footpads (FPs) of conventional mice [1], [2], whereas more prolific growth is attained in immunosuppressed rodents [3]–[6] and armadillos [7]. In these models bacterial multiplication is measured in terms of months to years.

Microscopic counting of acid fast bacilli (AFB) is used to enumerate *M. leprae* [2], [8], [9]. Although this method is considered the gold standard, it is time consuming, labor intensive and restricted with regard to specificity. It conveys the total number of bacteria present and does not distinguish between live and dead bacilli. More recently, a molecular technique for the enumeration of *M. leprae* based on real time PCR amplification of the repetitive element, RLEP, was described [10]. RLEP PCR had correlative results with microscopic counting and allowed for rapid and specific quantification of *M. leprae* from both mouse and armadillo tissues. Like microscopic counting, it does not provide absolute data on the viability of *M. leprae*. AFB counts and RLEP PCR yield viability information only indirectly, as bacterial numbers increase over time in a growing population.

For many years, the only way to truly assess the viability of a particular population of *M. leprae* was to inoculate serial dilutions of freshly harvested bacilli into the FPs of passage mice [11], [12]. Requiring hundreds of mice and at least a year of subculture to complete, this method, while effective, is highly impractical due to the length of time to obtain results, as well as the cost and numbers of experimental animals required. In an effort to simplify and expedite viability determination for *M. leprae*, a number of assays have been developed which investigate surrogate markers of viability, such as cell wall integrity or metabolism. These include measurement of morphologic index [13], [14], PGL-1 synthesis [15], [16], generation of intracellular ATP [16]–[20], palmitic acid oxidation in the BACTEC system [9], [21] and by radiorespirometry (RR) [9], [18], [20], [22], [23], and various viability stains [17], [24]–[27]. Currently, the most commonly used techniques are RR and the BacLight viability stain [27].

Improved methods for viability determination that are more sensitive and user friendly would be helpful for clinical and research purposes. A number of molecular assays have been proposed for determining *M. leprae* viability in environmental or clinical samples based on 16S ribosomal RNA [28]–[30] or messenger RNA (mRNA) ([31], [32]. Recently, a quantitative reverse transcription (qRT)-PCR based molecular assay was developed for *M. leprae* which used a gene transcript for *sodA* mRNA [30]. This assay was 100% specific for *M. leprae* and applicable as a viability indicator for bacilli recovered from short term macrophage cultures. Furthermore, the molecular data from the in vitro experiments showed a strong correlation with RR and BacLight viability staining.

The current studies built upon these principles with three objectives in mind. First, we sought to develop a sensitive and simple molecular assay which could accurately determine viability of *M. leprae* in infected tissue. Second, we examined the feasibility of eliminating the bacterial isolation steps and determining viability using nucleic acids isolated from ethanol-fixed FP tissues. Lastly, we evaluated the capacity of the molecular assays to monitor drug efficacy by comparing two leprosy drugs in a high bacterial burden, athymic mouse FP model. Results showed that when compared to the conventional methods of RR and BacLight viability staining, the *hsp18* and *esxA* qRT-PCR assays were sensitive and reliable biological indicators of *M. leprae* viability in tissues, and that concomitant host gene expression could be monitored from the same RNA preparations.

## Materials and Methods

### Ethics statement

These studies were performed under a scientific protocol reviewed and approved by the National Hansen's Disease Programs Institutional Animal Care and Use Committee (Assurance #A3032-01), and were conducted in accordance with all state and federal laws in adherence with PHS policy and as outlined in *The Guide to Care and Use of Laboratory Animals, Eighth Edition*.

### *M. leprae* and infection of mice

*M. leprae*, strain Thai-53, is maintained in athymic *nu/nu* mice through serial passage. Freshly harvested bacilli are stored at 4°C and used within 24 hours of harvest [9]. In this study, BALB/c and athymic *nu/nu* mice (Harlin Sprague-Dawley, Inc., Indianapolis, IN) were infected by inoculating each hind FP with 3×10^7^ *M. leprae* in 0.03 ml PBS (Irvine Scientific, Santa Ana, CA) [33], [34]. FPs were harvested on Day 1 post infection and at 4, 8, 12 and 17 weeks.

### Drug treatment

At 18 weeks post infection, groups of *M. leprae*-infected *nu/nu* mice were treated, by gavage, with rifampin (RMP, 10 mg/kg) or rifapentine (RPT, 10 mg/kg) emulsified in hydroxypropyl-β-cyclodextrin/L-α-phosphatidylcholine. All drugs were purchased from Sigma-Aldrich (St. Louis, MO). Each drug was administered as a single dose, five daily doses, or twenty daily doses (5 days per week for 4 weeks). Control mice were given vehicle only. FPs were harvested 1 month after completion of treatment for each regimen.

### Mouse FP harvest

The feet were disinfected with 70% ethanol and Betadine, the skin removed, and the FP tissue excised. Viable *M. leprae* were collected immediately from the right FPs. The left FP tissues were stored in 70% ethanol at −20°C until processed for DNA and RNA purification.

### *M. leprae* isolation

To harvest the bacilli, the right FP tissues were minced and gently homogenized in hand-held Tenbroeck tissue glass grinders (Fisher Scientific, Pittsburgh, PA) in 2.5 ml RPMI (Life Technologies, Grand Island, NY) containing 50 µg/ml ampicillin (Sigma-Aldrich) and trypsin. After incubation at 37°C for 15 minutes and slow speed centrifugation (100× g) for 1 minute to remove most of the tissue debris, the supernatants were pelleted (10,000× g for 30 minutes), resuspended, and sonicated in RPMI+10% FBS (Hyclone Laboratories, Logan, UT)+ampicillin. These bacterial suspensions were subjected to microscopic counting, BacLight viability staining, and RR.

### Microscopic counting of AFB

Three smears were prepared from each FP sample and counts of bacilli in twenty microscopic fields per smear were calculated to determine the number of AFB present in that particular FP [8]. Data are reported as mean +/− S.D. of 4–10 mice per group.

### Radiorespirometry (RR)

RR was performed as described previously [9]. Briefly, *M. leprae* from individual FPs were suspended in 1.0 ml of BACTEC 7H12B medium (Becton Dickinson, Franklin Lakes, NJ) in a 6 ml glass shorty vial (Wheaton Industries Inc., Millville, NJ) with a loosened cap. The vial was placed into a liquid scintillation vial with a 2″×4″ strip of Whatman #42 filter paper (Fisher Scientific) that had been soaked in Kodak concentrate I (Eastman Kodak Co., Rochester, NY) and dried. ^14^CO_2_ evolution was measured daily for seven days. Results were calculated as cpm ^14^CO_2_ per 10^6^ bacilli and reported as mean +/− S.D. of 4–10 mice per group.

### Viability staining

*M. leprae* from individual FPs were washed twice in sterile saline and stained using a BacLight Viability Staining Kit (Life Technologies) as previously described [27]. Briefly, the bacterial suspension was incubated for 15 minutes at room temperature in 6 µM Syto9 and 30 µM propidium iodine. The bacteria were washed with sterile saline and the pellet resuspended in 5% glycerol in saline. Five µl of the suspension was spread onto a slide, and viability was determined by counting the red and green bacilli, indicating dead and live bacteria, respectively, under a Nikon fluorescence microscope. The excitation/emission maxima are 480 nm/500 nm for Syto9 and 490 nm/635 nm for propidium iodide. Results are calculated as percent viability and reported as mean +/− S.D. of 4–10 mice per group.

### Purification of nucleic acids

RNA and DNA were purified from the left FPs using a previously described protocol [30]. Individual fixed FPs were removed from the ethanol, rehydrated, minced, suspended in 1.0 ml TRIzol reagent, and homogenized twice in FastRNA blue tubes using the FastPrep FP 24 instrument (MP Biomedicals, Solon, OH). Tubes were chilled on ice for 5 minutes, after which 200 µl of chloroform-isoamyl alcohol was added. After vortexing for 10 seconds and centrifugation at 700× g at 4°C for 5 minutes, the supernatants were transferred to new tubes, spun again at 14,000× g for 10 minutes, and the RNA collected from 300 µl of the aqueous phase. After incubation at −70°C overnight, the precipitated RNA was resuspended in 30 µl DEPC treated water, and contaminating DNA was removed using a Turbo DNA-free kit (Life Technologies). The purified RNA (150 µl) was stored at −70°C. DNA was purified by adding 100 µl of 10 mM Tris-EDTA and 150 µl of chloroform-isoamyl alcohol to the remaining aqueous phase and interphase material, homogenizing in the FastPrep 24 FP instrument twice, and centrifuging at 14,000× g for 10 minutes. The aqueous phase (200 µl) was precipitated with 5 M ammonium acetate and two volumes of cold ethanol, incubated at −70°C overnight, washed in 70% ethanol, dissolved in 30 µl 1× TE, and stored at −70°C.

### Reverse transcription

RNA from 3×10^3^ *M. leprae*, as determined from the number of RLEP genome equivalents from each specimen, was reverse transcribed. Titration experiments had shown that RNA from this number of *nu/nu* mouse-derived viable bacilli would consistently give a strong signal in the RT-PCR reactions. The RNA was converted to cDNA using an Advantage RT-for-PCR kit (Clontech, Mountain View, CA) consisting of reverse transcriptase, Advantage cDNA polymerase mix, and random hexamer primers at 42°C for 1 hour, 94°C for 5 minutes, and 4°C for 5 minutes. For mouse gene expression, 1 µg RNA was reverse transcribed to cDNA using the same conditions. Control for DNA contamination consisted of equivalent amounts of RNA, polymerase mix, and primers without the reverse transcriptase.

### Taqman

Molecular enumeration of *M. leprae* was determined using the purified DNA fraction from each specimen via Taqman technology using primers and a probe for a common region of the RLEP family of dispersed repeats in *M. leprae* as previously described [10]. Molecular viability of *M. leprae* was determined using the cDNA generated from the RNA fraction for each specimen and qRT-PCR. Primers and probes for each target sequence were designed using Primer Express 2.0 software (Life Technologies): *hsp*18 primers: forward – cgatcgggaaatgcttgc, reverse – cgagaaccagctgacgattg, probe - 6Fam-acaccgcgtggccgctcg; esxA primers: forward – ccgagggaataaaccatgca, reverse – cgtttcagccgagtgattga, probe - 6Fam-tgcttgcaccaggtcgccca. Five µl cDNA were added to the reaction mixture and real time PCR was performed using cycling conditions of 40 cycles of 60°C annealing, extension for 60 seconds, and 95°C denaturation for 15 seconds. PCR and data analyses for all assays were performed on a 7300 RealTime PCR System (Life Technologies). Results of the Taqman assays were applied to a standard curve generated by preparing 4-fold serial dilutions of a known number of *M. leprae*. Results were reported as mRNA equivalents for each gene transcript analyzed.

Mouse gene expression was evaluated utilizing cDNA and commercially available specific primer sets and probes for TNF, IFNγ, and CCL-2, and Universal Master Mix (Life Technologies). Data was analyzed by the ΔΔC_T_ method and expressed as a log-fold increase in expression over uninfected FPs. GAPDH was used to normalize for template variation. Results are reported as mean +/− S.D. of 4–10 mice per group.

### Statistical analyses

Data were analyzed using unpaired *t* tests or the non-parametric Mann-Whitney test and compared by group and within a group over time using SigmaPlot 12.0 software (Systat Software, Inc, Chicago, IL). Data was considered significant at P<0.05.

## Results

### Evaluation of *M. leprae* viability in mouse FPs using conventional assays

**Figure 1:**
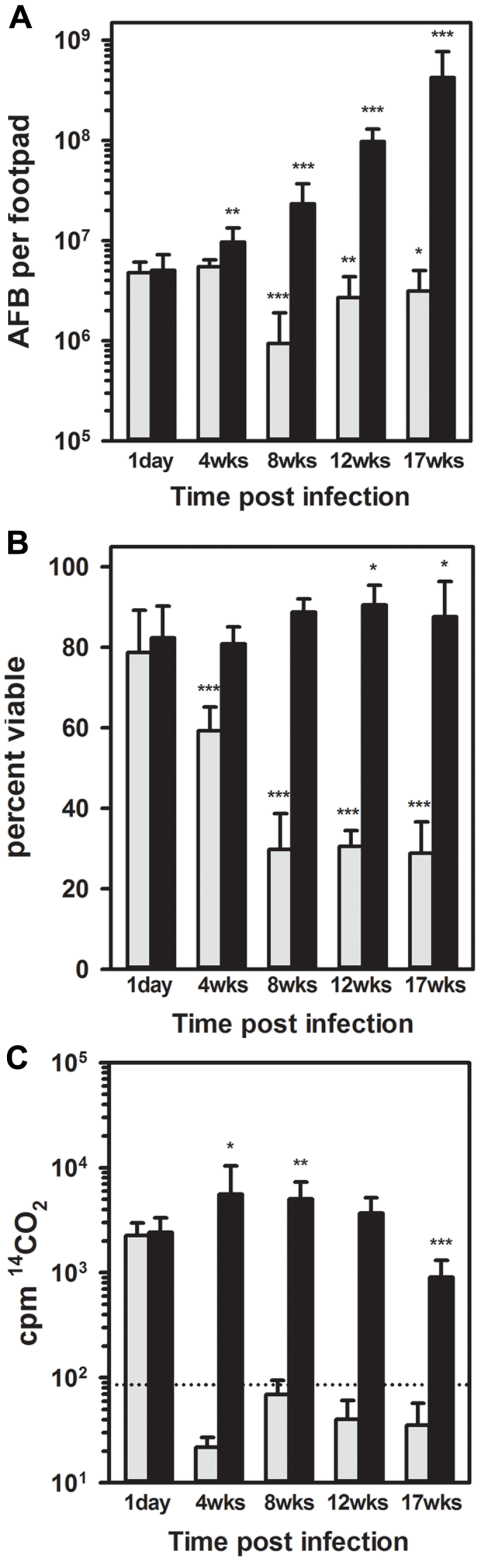
Enumeration and quantification of *M. leprae* in mouse FPs using conventional assays. BALB/c (gray bars) and athymic *nu/nu* (black bars) mice were infected in the FPs with 3×10^7^ *M. leprae*. Bacilli were harvested on day 1 and at 4, 8, 12, and 17 weeks post infection and enumerated by direct counting (A). Viability of *M. leprae* was determined by BacLight viability staining (B) and RR (C). Dotted line represents the lower detection limit of the assay. Bars represent mean and standard deviation for each group. The value at each time point was compared to its respective value at 1 day. * = probability of statistical significance (p)<0.05, ** = probability of statistical significance (p)<0.01, and *** = probability of statistical significance (p)<0.001.

Microscopic counting of AFB is shown in Figure 1A. On Day 1 post infection, 4.78×10^6^±1.30×10^6^ and 5.03×10^6^±2.23×10^6^ AFB were recovered from the BALB/c and *nu/nu* FPs, respectively. This recovery of approximately 16% of the inoculum is typical considering the architecture of the mouse FP and is consistent with previous reports [10], [35]. In the *nu/nu* FPs, the number of *M. leprae* steadily increased over the infection period reaching 4.25×10^8^±3.46×10^8^ by 17 weeks post infection (p<0.001). In contrast, the number of AFB in the BALB/c FPs remained steady at 4 weeks and then declined at 8 (p<0.001), 12 (p = 0.006), and 17 (p = 0.035) weeks.

The conventional viability assays, BacLight staining and RR, were performed on *M. leprae* isolated from the BALB/c and *nu/nu* mouse FPs throughout the infection period to determine its viability. Using the BacLight staining method (Figure 1B), *M. leprae* from BALB/c and *nu/nu* FPs were 78.71±10.53 and 82.34±7.87 percent viable, respectively, on Day 1. Percent viability increased to 90.51±4.90 in the *nu/nu* FPs by 12 weeks (p = 0.029) post infection. In BALB/c mice, *M. leprae* viability decreased to 60.72±7.16 percent by 4 weeks (p<0.001) and to 29.79±8.90 percent by 8 weeks (p<0.001) post infection. Percent viability held at this level for the remainder of the infection period.

Using the RR assay (Figure 1C), *M. leprae* viability reported as ^14^CO_2_ generated per 10^6^ bacilli showed a slight increase by 4weeks (p = 0.049) in the *nu/nu* mice. In contrast, *M. leprae* from BALB/c FPs exhibited a 1.5 log decline in metabolic activity by 4weeks post infection (p<0.001).

### Molecular evaluation of *M. leprae* viability in mouse FPs

**Figure 2:**
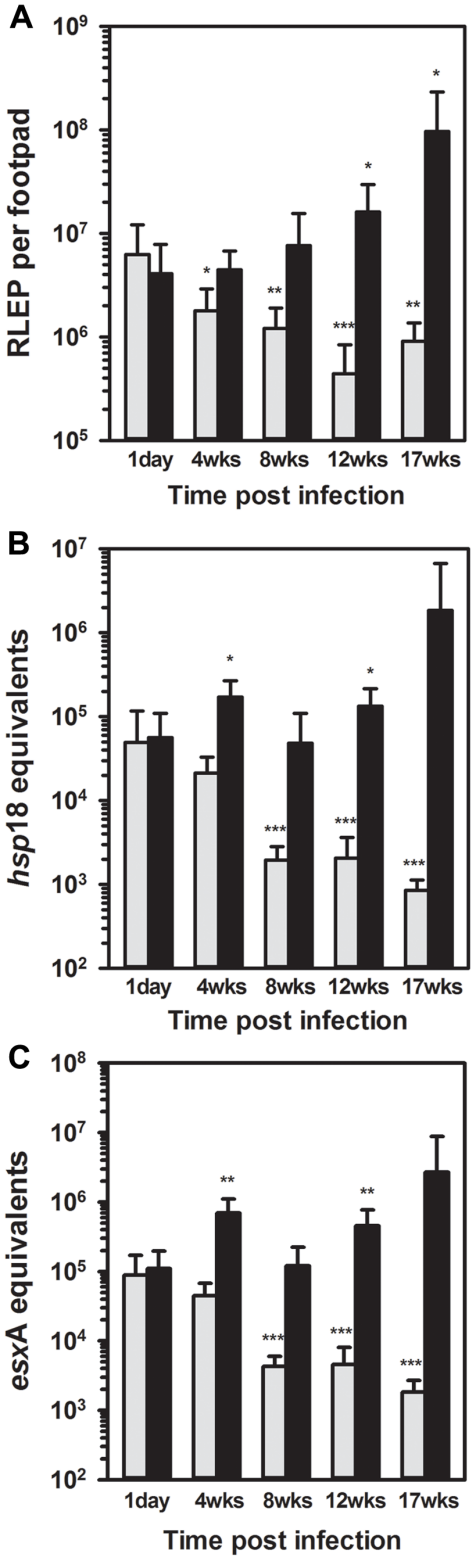
Enumeration and quantification of *M. leprae* in mouse FPs by molecular assays. BALB/c (gray bars) and athymic *nu/nu* (black bars) mice were infected in with 3×10^7^ *M. leprae*. FP tissues were fixed in 70% ethanol on day 1 and at 4, 8, 12, and 17 weeks post infection. DNA and RNA were purified using a FastPrep protocol. *M. leprae* were enumerated by RLEP PCR on the DNA fraction (A). cDNA was prepared from an RNA equivalent of 3×10^3^ *M. leprae* for determination of viability by *hsp18* (B) and *esxA* (C) qRT-PCR. Bars represent mean and standard deviation for each group. The value at each time point was compared to its respective value at 1 day. * = probability of statistical significance (p)<0.05, ** = probability of statistical significance (p)<0.01, and *** = probability of statistical significance (p)<0.001.

RLEP enumeration, performed on DNA purified from the left FP tissues, demonstrated that 6.27×10^6^±5.88×10^6^ *M. leprae* were recovered from BALB/c FPs and 4.07×10^6^±3.78×10^6^ were recovered from *nu/nu* FPs on Day 1 (Figure 2A). An initial lag phase was evident in the *nu/nu* FPs yet growth reached 9.70×10^7^±1.35×10^8^ (p = 0.015) by 17 weeks post infection. In the BALB/c FPs, there was a significant decrease in the number of *M. leprae* at 4 (p = 0.022), 8 (p = 0.003), 12 (p<0.001) and 17 (p = 0.002) weeks.

*hsp18* and *esxA* qRT-PCR assays yielded strong signals when evaluated for use as indicators of *M. leprae* viability in our models. In both assays, expression of the transcripts was maintained in the *nu/nu* FPs on the order of ∼10^5^ *hsp18* (Figure 2B) or *esxA* (Figure 2C) equivalents per 3×10^3^ *M. leprae* over the course of infection. In contrast, these assays demonstrated a sharp decline in viability in the BALB/c FPs by 8 weeks to 1.95×10^3^±8.76×10^2^ for *hsp18* (p<0.001) and 4.30×10^3^±1.69×10^3^ for *esxA* (p<0.001), and remained on the order of 10^3^ equivalents for the remainder of the infection period.

### RMP and RPT Treatment of *M. leprae*-infected mice

The conventional and molecular assays were compared in a multibacillary FP model for their capacity to monitor drug efficacy. Athymic *nu/nu* mice were infected with 3×10^7^ *M. leprae*. At 18 weeks post infection, rifampin or rifapentine (each at 10 mg/kg) were administered to groups of mice for 1 treatment (1×), 5 daily treatments (5×) or 20 doses at 5 days per week for 4 weeks (20×). FPs were harvested 1 month post treatment for each regimen.

**Figure 3:**
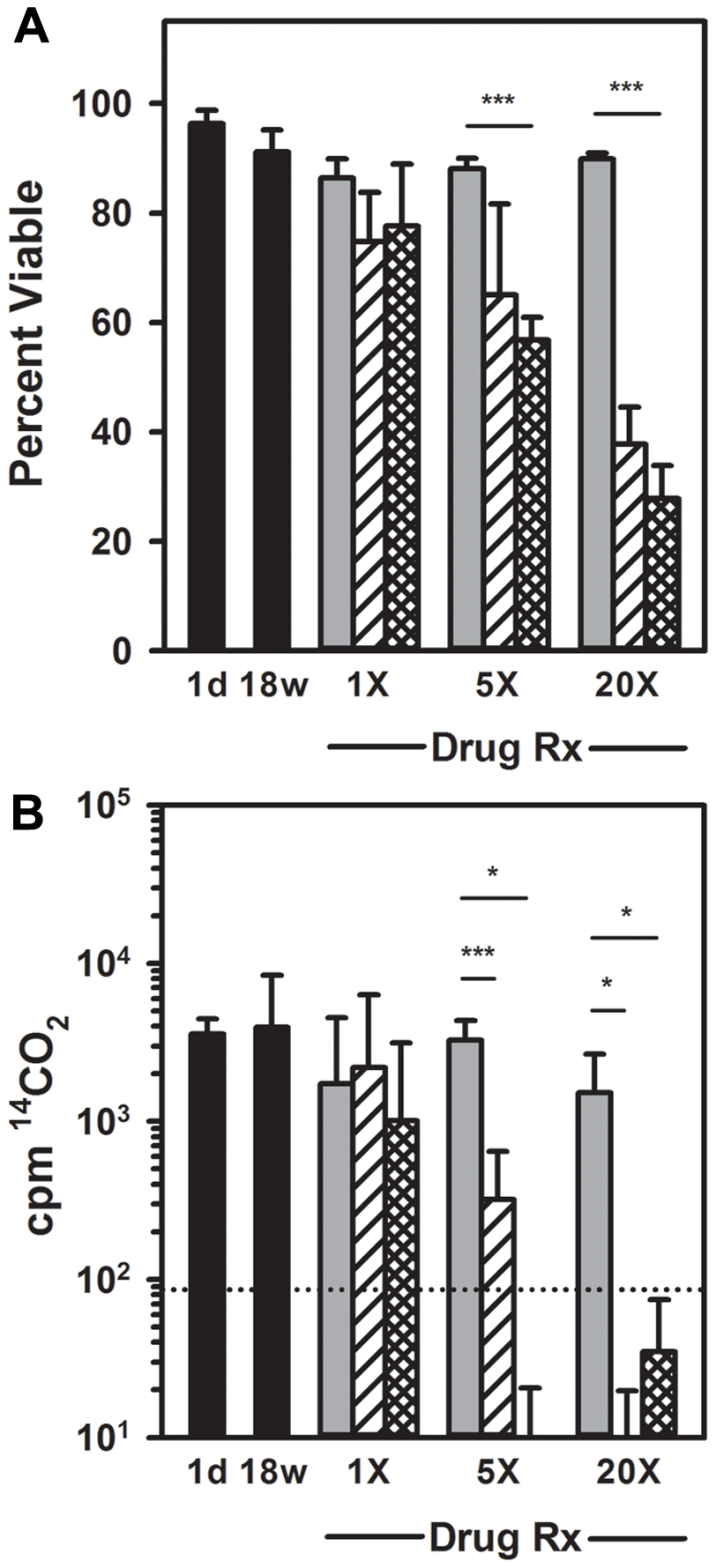
Enumeration and quantification using conventional assays of *M. leprae* from RMP and RPT treated mice. Athymic *nu/nu* (black bars) mice were infected in the FPs with 3×10^7^ *M. leprae*. At 18 weeks post infection, groups of mice were treated with 1 dose (1×), 5 daily doses (5×), or 20 daily doses (20×) of RMP (striped bars) or RPT (crosshatched bars), each at 10 mg/kg. Control mice received vehicle alone (gray bars). One month after the last dose of each regimen, bacilli were harvested and viability of *M. leprae* was determined by BacLight viability staining (A) and RR (B). Dotted line represents the lower detection limit of the assay. Bars represent mean and standard deviation for each group. * = probability of statistical significance (p)<0.05, and *** = probability of statistical significance (p)<0.001.

Results of the conventional viability assays are shown in Figure 3. One treatment with rifampin or rifapentine did not decrease *M. leprae* viability when measured by BacLight staining (Figure 3A) or RR (Figure 3B). Rifapentine reduced *M. leprae* viability to 56.83±4.11 percent (p<0.001) after 5 daily treatments and to 27.78±6.05 percent (p<0.001) with 20 doses as measured by BacLight staining (Figure 3A). Twenty doses of rifampin decreased viability to 37.71±6.79 percent (p = 0.057). Rifampin was more effective when assessed using the RR assay (Figure 3B) and decreased *M. leprae* metabolic activity by approximately 1 log (p<0.001) after 5 treatments. Rifapentine treatment reduced metabolism >2 log (p = 0.016). Both rifampin and rifapentine were active at 20× (p = 0.016).

**Figure 4:**
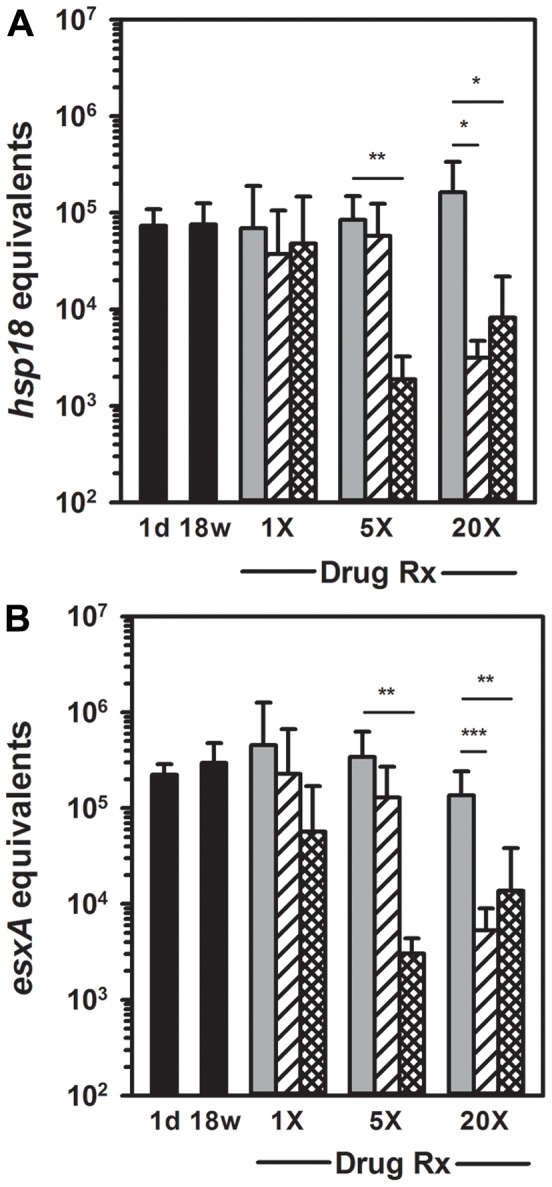
Enumeration and quantification via molecular assays of *M. leprae* from RMP and RPT treated mice. Athymic *nu/nu* (black bars) mice were infected in the FPs with 3×10^7^ *M. leprae*. At 18 weeks post infection, groups of mice were treated with 1 dose (1×), 5 daily doses (5×), or 20 daily doses (20×) of RMP (striped bars) or RPT (crosshatched bars), each at 10 mg/kg. Control mice received vehicle alone (gray bars). One month after the last dose of each regimen, FP tissues were fixed in 70% ethanol. DNA and RNA were purified using a FastPrep protocol. *M. leprae* were enumerated by RLEP PCR on the DNA fraction, and cDNA was prepared from an RNA equivalent of 3×10^3^ *M. leprae* for determination of viability by *hsp18* (A) and *esxA* (B) qRT-PCR. Bars represent mean and standard deviation for each group. * = probability of statistical significance (p)<0.05, ** = probability of statistical significance (p)<0.01, and *** = probability of statistical significance (p)<0.001. 5× RPT vs. 20× RPT: (p) = 0.396 for *hsp18*; (p) = 0.569 for *esxA*.

In agreement with the conventional viability assays, neither rifampin nor rifapentine at a single dose decreased *M. leprae* viability when assessed by *hsp18* (Figure 4A) or *esxA* (Figure 4B) qRT-PCR. Control FPs expressed ∼10^5^ *hsp18* or *esxA* equivalents per 3×10^3^ *M. leprae*. Five doses of rifapentine reduced this to 1.88×10^3^±1.37×10^3^ *hsp18* (p = 0.001) and 3.04×10^3^±1.33×10^3^ *esxA* (p = 0.001) equivalents, while 5× rifampin showed no significant decrease in expression in either assay. However, these molecular assays demonstrated that both rifampin and rifapentine were highly effective at 20 doses when measured by *hsp18* expression (p = 0.016 and p = 0.019, respectively) or *esxA* expression (p<0.001 and p = 0.009, respectively).

### Host gene expression in *M. leprae*-infected FPs

The RNA preparations were also examined for cytokine and chemokine expression. As shown in Table 1, high levels of TNF were expressed by Day 1in the FPs of both strains of mice. TNF expression increased in BALB/c FPs by 8 weeks (p = 0.031) but decreased in *nu/nu* FPs (p = 0.028). Little or no IFNγ was expressed by either strain on Day 1, but expression increased by >2 log in BALB/c (p<0.001). IFNγ expression also increased in *nu/nu* FPs (p = 0.005) but not to the extent of BALB/c. Similar levels of CCL-2 were expressed by both strains at both time points.

**Table 1:** Host gene expression in *M. leprae*-infected mouse FPs.

Strain/Time^a^	TNF^b^	p	IFNγ^b^	p	CCL-2^b^	p
BALB/c						
T0	1.65±0.40		0.44±0.36		1.10±0.34	
T+8 weeks	2.04±0.30	0.031	2.21±0.31	<0.001	1.40±0.88	NS
*nu/nu*						
T0	1.53±0.46		0.00±0.27		0.93±0.42	
T+8 weeks	1.16±0.26	0.028	0.50±0.41	0.005	2.12±1.08	NS

a Mice were infected with 3×10^7^ *M. leprae*. FP tissues were harvested and fixed in 70% ethanol on Day 1 and at 8 weeks post infection. RNA was purified using a FastPrep protocol, and cDNA was prepared from 1 µg RNA and subjected to PCR for TNF, IFNγ and CCL-2.

b Data was normalized to GAPDH and expressed as a log-fold increase over uninfected FPs.

## Discussion

The lack of an *in vitro* cultivation system for *M. leprae* has made determination of its viability extremely difficult in experimental models of the disease and in human lesions because current techniques require large numbers of purified, viable bacteria. This restricts investigation into the pathogenicity of *M. leprae* as well as the experimental testing of novel drugs for the treatment of leprosy. Therefore, the purpose of this study was to develop a simple and sensitive molecular assay for determining *M. leprae* viability directly from infected tissues. Two *M. leprae*-specific qRT-PCR assays based on the expression levels of *esxA* and *hsp18* were developed and tested in the mouse FP model using both immunocompetent and immunocompromised mice. These qRT-PCR assays could detect high viability in the athymic *nu/nu* FP as well as killing of *M. leprae* by the host immune system in the BALB/c mouse, or by antimicrobial treatment of *nu/nu* mice having highly multibacillary FPs. The RNA preparations were also successfully used for detection of host cytokine expression.

The hypothesis tested in this study was that viability is related to the expression of specific genes; therefore, monitoring a specific *M. leprae* gene transcript(s) by qRT-PCR should provide a simple and sensitive assay for determining its viability. Molecular methods have been developed to ascertain the viability of several infectious organisms [36]. Early studies used levels of ribosomal RNA as a marker of viability [37]–[40]. However, its long half-life and inconsistent retention made it somewhat less accurate, especially for short term experimentation. Because of its relatively short half-life, mRNA has been used successfully as a viability indicator for a number of pathogens [41]–[43] including *M. tuberculosis* [44]–[46]. The choice of transcript was an important consideration in all of these studies, not only for sensitivity but also for its expression under a variety of circumstances. In addition, different viability assays have varying abilities to differentiate cell death, which is often highly dependent on how the organism is killed and how cell death is defined [41], [47].

Initially we tested the expression profiles of several *M. leprae* genes as potential indicators of viability in our system. These included: *sodA*, encoding superoxide dismutase A and which was used successfully in the *M. leprae*-infected macrophage cultures [30]; *gap*, encoding glyceraldehyde-3-phosphate dehydrogenase; ML2138C, encoding a probable transmembrane protein; *hsp18*, encoding the 18 kD heat shock protein; and *esxA*, encoding the ESAT-6 protein. These transcripts were chosen based on their high expression levels in DNA microarray experiments at 6 months post infection in the athymic *nu/nu* FP model [48]. However, *sodA*, *gap*, and ML2138C genes were not transcribed in sufficient quantities at the early time points in the FP models and therefore did not possess the requisite sensitivity (data not shown). In contrast, the *hsp18* and *esxA*-based assays were highly expressed in both immunocompetent and immunosuppressed mice. Moreover, they were both able to accurately determine loss of viability when using two different methods of bacterial killing, i.e. immunologically mediated and anti-microbial drugs, which strengthens the validity and usefulness of these assays.

In the present study, athymic *nu/nu* and BALB/c mouse FPs were inoculated with a relatively high dose of *M. leprae*. We chose this dose for infection because, as an immunizing dose in BALB/c immunocompetent mice, it would be recognized and killed in the first 1–2 months of infection; yet, in the immunocompromised athymic *nu/nu* mice the inoculum would continue to grow. When inoculated with fewer *M. leprae*, (e.g., 10^3^ to 10^4^), the maximum growth attained in an immunocompetent mouse FP is approximately 10^6^ bacilli [1]. This growth plateau, which is seen at approximately 6 months post infection, is due to death of the bacilli by the immune response. Once bacterial numbers peak, viability decreases with a half-time of loss of 25 days [12]. Using the *hsp18* and *esxA* qRT-PCR assays, a strong signal could be obtained with RNA from 3×10^3^ *M. leprae*.

Over the years a variety of techniques have been developed in an attempt to circumvent the inability to culture this organism. Each assay measures different aspects of *M. leprae* viability and has provided insights into its unique properties. For our conventional viability determinations, we used RR [9], [18], [20], [22], [23] and BacLight viability staining [27]. RR measures the oxidation of ^14^C-palmitic acid to ^14^CO_2_; thus, viability is defined in terms of the metabolic activity of the bacterial population. Viability staining uses fluorescent dyes that bind nucleic acids, with one dye that can penetrate cell membranes and one that cannot. A differential staining pattern is exhibited by live bacteria with intact cell membranes versus dead bacteria with damaged membranes. Thus, viability in this assay is assessed in terms of membrane integrity of individual bacteria. While these assays work very well for in vitro viability determinations, and for ex vivo use, they also have their limitations. Both require immediate, labor intensive purification of *M. leprae* and processing of samples as the bacilli rapidly lose viability once removed from the host. The bacterial population must be relatively clean and free of most host tissue debris to obtain clear results, especially for the BacLight assay. For RR, a minimum of 10^6^ organisms are required for accurate readings. Furthermore, matters regarding the use of radioisotopes, including worker safety and waste disposal, must be considered. Although valuable in the laboratory, these conditions make these assays impractical for evaluating *M. leprae* viability in biopsy specimens in clinical or field settings where the reagents and equipment required for bacterial isolation and viability assessment are not readily available.

Therefore, in conjunction with the development of the molecular assays, we evaluated the feasibility of eliminating the bacterial isolation steps and assessing viability on nucleic acids purified directly from ethanol-fixed FP tissue. Our success with this protocol certainly indicates potential for use with biopsy specimens in the clinic or field. Although we have not evaluated formalin-fixed paraffin-embedded tissue (FFPE) for RT-PCR, Su, et al. [49] have shown that ethanol fixation for samples slated for RT-PCR is far superior to FFPE and that these tissues can be stored for weeks. Our unpublished results show that RNA can be preserved for months in 70% ethanol at room temperature. Thus, tissues can be easily fixed and stored in 70% ethanol for transport back to the laboratory for processing [50], [51].

A potential application for these molecular viability assays is in the monitoring of treatment. Lesions of multibacillary patients often contain numerous bacilli even years after completion of multidrug therapy, inciting concern over the possibility of inadequate treatment, lack of compliance, or drug resistance. Our studies using well defined models, i.e. immunocompetent and athymic *nu/nu* mice infected with a well characterized inoculum [9] of known viability and duration of infection, have allowed characterization of the optimum parameters for use of the *hsp18* and *esxA* transcripts as viability indicators for *M. leprae* in tissues. Further studies, of course, must be done to determine whether these parameters will suffice for assessing viability of *M. leprae* in patient samples.

RMP is a very effective anti-leprosy drug and an integral part of the WHO multidrug regimen [52]. Early investigations with RMP found it to be more rapidly bactericidal than dapsone [53], [54], with reports that even a single treatment of patients rendered *M. leprae* non-infectious for mice [55]. Similarly, treatment of *M. leprae*-infected immunocompetent mice with a single dose of RMP had a significant bactericidal effect [54], [56]–[58]. Subsequently, several investigators examined various therapeutic regimens, with and without RMP, using *M. leprae* infection of athymic *nu/nu* mice. The *nu/nu* model removes the likely contribution of the immune system to aid the drug therapy in its bactericidal effects and treatment of a high bacterial burden, “lepromatous” infection can be tested. The efficacy of rifampin in this model has been variable [59]–[63]. Early studies showed that single [60] or intermittent [59] dosing with RMP was not effective and that 99.99% killing was not achieved with each dose.

In a large, well-controlled trial designed to determine the effectiveness of single dose rifampicin to prevent leprosy in close contacts in a high endemic area, Moet, et al. [64] found a 57% reduction in the overall incidence of leprosy in the treatment group at 2 years. This efficacy was maintained but not improved at 4 and 6 years of follow-up [64], [65]. However, when they evaluated subgroups of contacts, chemoprophylaxis with single dose rifampicin was less effective in contacts of patients with multibacillary disease and in contacts that were seropositive for PGL-1. They postulated that the bacillary load in these contacts at the time of treatment may have already been too high to be eliminated.

In our present study, the purpose of the RMP and RPT treatments was to validate the molecular viability assays, and we used a high bacterial burden, lepromatous model. Neither a single dose nor 5 daily doses of RMP were effective when measured by either BacLight staining or the molecular assays, although a decrease in metabolic activity was detected by RR after 5 doses. Treatment with 20 daily doses, however, showed strong inhibition in all assays. RPT, in contrast was effective at 5 daily doses, likely due to its longer retention and more potent anti-mycobacterial activity [58], [66]–[69]. Altogether, these collective findings underscore the adjunctive role played by immunity in successful chemotherapy and emphasize the issues which must be considered when treating the susceptible host.

An added bonus to monitoring gene expression in situ is that host gene expression can be determined using the same RNA preparations. If using the conventional assays in our experimental studies, separate groups of mice must be employed for host gene expression and *M. leprae* enumeration and viability determinations. With the molecular assays, both host and bacterial expression can be determined using the same RNA samples, thereby greatly reducing the number of animals required for an experiment. Likewise, successful application of these techniques in the clinical setting could enable the monitoring of *M. leprae* viability and correlation with the immune response. A particular need would be during the treatment of reactional episodes, an aspect of leprosy which is still poorly understood.

In conclusion, these results show that the *hsp18* and *esxA* qRT-PCR assays are reliable indicators for determining *M. leprae* viability in tissue specimens. Their sensitivity and simplicity make them useful for initial in vivo drug screening. Moreover, their ease of use makes them attractive for potential application in clinical and field settings, and both *M. leprae* viability as well as host responses during treatment can be monitored.

## References

[1] Shepard CC (1962) Multiplication of *Mycobacterium leprae* in the foot-pad of the mouse. Int J Lepr 30: 291–306. 13976752

[2] Levy L, Ji B (2006) The mouse foot-pad technique for cultivation of *Mycobacterium leprae* . Lepr Rev 77: 5–24. 16715686

[3] Rees RJ, Waters MF, Weddell AG, Palmer E (1967) Experimental lepromatous leprosy. Nature 215: 599–602. 4860661 10.1038/215599a0

[4] Colston MJ, Hilson GR (1976) Growth of *Mycobacterium leprae* and *M. marinum* in congenitally athymic (nude) mice. Nature 262: 399–401. 785274 10.1038/262399a0

[5] Dawson PJ, Colston MJ, Fieldsteel AH (1983) Infection of the congenitally athymic rat with *Mycobacterium leprae* . Int J Lepr Other Mycobact Dis 51: 336–346. 6685695

[6] Chehl S, Ruby J, Job CK, Hastings RC (1983) The growth of *Mycobacterium leprae* in nude mice. Lepr Rev 54: 283–304. 6230496 10.5935/0305-7518.19830035

[7] Kirchheimer WF, Storrs EE (1971) Attempts to establish the armadillo (*Dasypus novemcinctus linn*.) as a model for the study of leprosy. I. Report of lepromatoid leprosy in an experimentally infected armadillo. Int J Lepr Other Mycobact Dis 39: 693–702. 4948218

[8] Shepard CC, McRae DH (1968) A method for counting acid-fast bacteria. Int J Lepr Other Mycobact Dis 36: 78–82. 4869698

[9] Truman RW, Krahenbuhl JL (2001) Viable *M. leprae* as a research reagent. Int J Lepr Other Mycobact Dis 69: 1–12. 11480310

[10] Truman RW, Andrews PK, Robbins NY, Adams LB, Krahenbuhl JL, et al (2008) Enumeration of *Mycobacterium leprae* using real-time PCR. PLoS Negl Trop Dis 2: E328. 18982056 10.1371/journal.pntd.0000328 PMC2570796

[11] Colston MJ, Hilson GR, Banerjee DK (1978) The “proportional bactericidal test”: A method for assessing bactericidal activity in drugs against *Mycobacterium leprae* in mice. Lepr Rev 49: 7–15. 347206

[12] Welch TM, Gelber RH, Murray LP, Ng H, O'Neill SM, et al (1980) Viability of *Mycobacterium leprae* after multiplication in mice. Infect Immun 30: 325–328. 7002795 10.1128/iai.30.2.325-328.1980 PMC551313

[13] Shepard CC, McRae DH (1965) M*ycobacterium leprae*: Viability at 0 degrees C, 31 degrees C, and during freezing. Int J Lepr 33: 316–323. 5322769

[14] Kaur S, Minocha YC, Sengupta U, Naik S (1975) A comparative evaluation of bacteriologic and morphologic indices of *Mycobacterium leprae* in skin, lymph node, bone marrow, nerve and muscle. Int J Lepr Other Mycobact Dis 43: 55–57. 1099019

[15] Ramasesh N, Hastings RC, Krahenbuhl JL (1987) Metabolism of *Mycobacterium leprae* in macrophages. Infect Immun 55: 1203–1206. 3552993 10.1128/iai.55.5.1203-1206.1987 PMC260491

[16] Franzblau SG, Harris EB (1988) Biophysical optima for metabolism of *Mycobacterium leprae* . J Clin Microbiol 26: 1124–1129. 3290244 10.1128/jcm.26.6.1124-1129.1988 PMC266546

[17] Katoch VM, Katoch K, Ramanathan U, Sharma VD, Shivannavar CT, et al (1989) Effect of chemotherapy on viability of *Mycobacterium leprae* as determined by ATP content, morphological index and FDA-EB fluorescent staining. Int J Lepr Other Mycobact Dis 57: 615–621. 2476522

[18] Ramasesh N, Adams LB, Franzblau SG, Krahenbuhl JL (1991) Effects of activated macrophages on *Mycobacterium leprae* . Infect Immun 59: 2864–2869. 1908824 10.1128/iai.59.9.2864-2869.1991 PMC258106

[19] Gupta UD, Katoch K, Natarajan M, Sharma VD, Sharma RK, et al (1997) Viability determination of *M. leprae*: Comparison of normal mouse foot pad and bacillary ATP bioluminescence assay. Acta Leprol 10: 209–212. 9447254

[20] Agrawal VP, Shetty VP (2007) Comparison of radiorespirometric budemeyer assay with ATP assay and mouse foot pad test in detecting viable *Mycobacterium leprae* from clinical samples. Indian J Med Microbiol 25: 358–363. 18087085

[21] Franzblau SG (1989) Drug susceptibility testing of *Mycobacterium leprae* in the BACTEC 460 system. Antimicrob Agents Chemother 33: 2115–2117. 2694952 10.1128/aac.33.12.2115 PMC172831

[22] Franzblau SG (1988) Oxidation of palmitic acid by *Mycobacterium leprae* in an axenic medium. J Clin Microbiol 26: 18–21. 3125213 10.1128/jcm.26.1.18-21.1988 PMC266168

[23] Agrawal VP, Shetty VP (2007) Comparison of the mouse foot pad test with a Buddemeyer type radiorespirometric assay in detecting viable *Mycobacterium leprae* in human lesional biopsies. Indian J Dermatol Venereol Leprol 73: 384–388. 18032855 10.4103/0378-6323.37054

[24] Kvach JT, Munguia G, Strand SH (1984) Staining tissue-derived *Mycobacterium leprae* with fluorescein diacetate and ethidium bromide. Int J Lepr Other Mycobact Dis 52: 176–182. 6202650

[25] Harshan KV, Prasad HK, Chopra NK, Mishra RS, Gogiya P, et al (1987) Comparison of radiometric macrophage assay and fluorescein diacetate/ethidium bromide staining for evaluation of *M. leprae* viability. Int J Lepr Other Mycobact Dis 55: 316–321. 2439621

[26] Bhagria A, Mahadevan PR (1987) A rapid method for viability and drug sensitivity of *Mycobacterium leprae* cultured in macrophages and using fluorescein diacetate. Indian J Lepr 59: 9–19. 2440960

[27] Lahiri R, Randhawa B, Krahenbuhl J (2005) Application of a viability-staining method for *Mycobacterium leprae* derived from the athymic (*nu/nu*) mouse foot pad. J Med Microbiol 54: 235–242. 15713606 10.1099/jmm.0.45700-0

[28] Lavania M, Katoch K, Katoch VM, Gupta AK, Chauhan DS, et al (2008) Detection of viable *Mycobacterium leprae* in soil samples: insights into possible sources of transmission of leprosy. Infect Genet Evol 8: 627–631. 18599381 10.1016/j.meegid.2008.05.007

[29] Sharma R, Lavania M, Katoch K, Chauhan DS, Gupta AK, et al (2008) Development and evaluation of real-time RT-PCR assay for quantitative estimation of viable *Mycobacterium leprae* in clinical samples. Indian J Lepr 80: 315–321. 20329380

[30] Martinez AN, Lahiri R, Pittman TL, Scollard D, Truman R, et al (2009) Molecular determination of *Mycobacterium leprae* viability by use of real-time PCR. J Clin Microbiol 47: 2124–2130. 19439537 10.1128/JCM.00512-09 PMC2708532

[31] Patel BK, Banerjee DK, Butcher PD (1993) Determination of *Mycobacterium leprae* viability by polymerase chain reaction amplification of 71-kDa heat-shock protein mRNA. J Infect Dis 168: 799–800. 8354932 10.1093/infdis/168.3.799

[32] Chae GT, Kim MJ, Kang TJ, Lee SB, Shin HK, et al (2002) DNA-PCR and RT-PCR for the 18-kDA gene of *Mycobacterium leprae* to assess the efficacy of multi-drug therapy for leprosy. J Med Microbiol 51: 417–422. 11990494 10.1099/0022-1317-51-5-417

[33] Hagge DA, Marks VT, Ray NA, Dietrich MA, Kearney MT, et al (2007) Emergence of an effective adaptive cell mediated immune response to *Mycobacterium leprae* is not impaired in reactive oxygen intermediate-deficient mice. FEMS Immunol Med Microbiol 51: 92–101. 17645529 10.1111/j.1574-695X.2007.00282.x

[34] Adams LB, Pena MT, Sharma R, Hagge DA, Schurr E, et al (2012) Insights from animal models on the immunogenetics of leprosy: A review. Mem Inst Oswaldo Cruz 107 Suppl 1: 197–208. 23283472 10.1590/s0074-02762012000900028

[35] Levy L, Moon N, Murray LP, O'Neill SM, Gustafson LE, et al (1974) Studies of the mouse foot pad technic for cultivation of *Mycobacterium leprae*. 1. Fate of inoculated organisms. Int J Lepr Other Mycobact Dis 42: 165–173. 4609928

[36] Keer JT, Birch L (2003) Molecular methods for the assessment of bacterial viability. J Microbiol Methods 53: 175–183. 12654489 10.1016/s0167-7012(03)00025-3

[37] McKillip JL, Jaykus LA, Drake M (1998) rRNA stability in heat-killed and UV-irradiated enterotoxigenic *Staphylococcus aureus* and *Escherichia coli* O157:H7. Appl Environ Microbiol 64: 4264–4268. 9797275 10.1128/aem.64.11.4264-4268.1998 PMC106637

[38] Villarino A, Bouvet OM, Regnault B, Martin-Delautre S, Grimont PAD (2000) Exploring the frontier between life and death in *Escherichia coli*: Evaluation of different viability markers in live and heat- or UV-killed cells. Res Microbiol 151: 755–768. 11130866 10.1016/s0923-2508(00)01141-4

[39] Bergin PF, Doppelt JD, Hamilton WG, Mirick GE, Jones AE, et al (2010) Detection of periprosthetic infections with use of ribosomal RNA-based polymerase chain reaction. J Bone Joint Surg Am 92: 654–663. 20194324 10.2106/JBJS.I.00400 PMC2827826

[40] Aarthi P, Bagyalakshmi R, Therese KL, Madhavan HN (2013) Development of a novel reverse transcriptase polymerase chain reaction to determine the gram reaction and viability of bacteria in clinical specimens. Microbiol Res 168: 497–503. 23602123 10.1016/j.micres.2013.03.005

[41] Sheridan GE, Masters CI, Shallcross JA, Mackey BM (1998) Detection of mRNA by reverse transcription-PCR as an indicator of viability in *Escherichia coli* cells. Appl Environ Microbiol 64: 1313–1318. 9546166 10.1128/aem.64.4.1313-1318.1998 PMC106147

[42] Baque RH, Gilliam AO, Robles LD, Jakubowski W, Slifko TR (2011) A real-time RT-PCR method to detect viable *Giardia lamblia* cysts in environmental waters. Water Res 45: 3175–3184. 21501854 10.1016/j.watres.2011.03.032

[43] Alum A, Sbai B, Asaad H, Rubino JR, Khalid Ijaz M (2012) ECC-RT-PCR: A new method to determine the viability and infectivity of *Giardia* cysts. Int J Infect Dis 16: E350–353. 22390842 10.1016/j.ijid.2012.01.004

[44] Hellyer TJ, Desjardin LE, Hehman GL, Cave MD, Eisenach KD (1999) Quantitative analysis of mRNA as a marker for viability of *Mycobacterium tuberculosis* . J Clin Microbiol 37: 290–295. 9889206 10.1128/jcm.37.2.290-295.1999 PMC84288

[45] Desjardin LE, Perkins MD, Wolski K, Haun S, Teixeira L, et al (1999) Measurement of sputum *Mycobacterium tuberculosis* messenger RNA as a surrogate for response to chemotherapy. Am J Respir Crit Care Med 160: 203–210. 10390401 10.1164/ajrccm.160.1.9811006

[46] Li L, Mahan CS, Palaci M, Horter L, Loeffelholz L, et al (2010) Sputum *Mycobacterium tuberculosis* mRNA as a marker of bacteriologic clearance in response to antituberculosis therapy. J Clin Microbiol 48: 46–51. 19923475 10.1128/JCM.01526-09 PMC2812283

[47] Villarino A, Rager MN, Grimont PA, Bouvet OM (2003) Are UV-induced nonculturable *Escherichia coli* K-12 cells alive or dead? Eur J Biochem 270: 2689–2695. 12787036 10.1046/j.1432-1033.2003.03652.x

[48] Williams DL, Torrero M, Wheeler PR, Truman RW, Yoder M, et al (2004) Biological implications of *Mycobacterium leprae* gene expression during infection. J Mol Microbiol Biotechnol 8: 58–72. 15741741 10.1159/000082081

[49] Su JM, Perlaky L, Li XN, Leung HC, Antalffy B, et al (2004) Comparison of ethanol versus formalin fixation on preservation of histology and RNA in laser capture microdissected brain tissues. Brain Pathol 14: 175–182. 15193030 10.1111/j.1750-3639.2004.tb00050.x PMC8096012

[50] Fiallo P, Williams DL, Chan GP, Gillis TP (1992) Effects of fixation on polymerase chain reaction detection of I. J Clin Microbiol 30: 3095–3098. 1452690 10.1128/jcm.30.12.3095-3098.1992 PMC270594

[51] Williams DL, Oby-Robinson S, Pittman TL, Scollard DM (2003) Purification of *Mycobacterium leprae* RNA for gene expression analysis from leprosy biopsy specimens. Biotechniques 35: 534–531, 534-536, 538, 540-531. 14513559 10.2144/03353st07

[52] Scollard DM, Adams LB, Gillis TP, Krahenbuhl JL, Truman RW, et al (2006) The continuing challenges of leprosy. Clin Microbiol Rev 19: 338–381. 16614253 10.1128/CMR.19.2.338-381.2006 PMC1471987

[53] Rees RJ, Pearson JM, Waters MF (1970) Experimental and clinical studies on rifampicin in treatment of leprosy. Br Med J 1: 89–92. 4903972 10.1136/bmj.1.5688.89 PMC1699176

[54] Shepard CC, Levy L, Fasal P (1972) Rapid bactericidal effect of rifampin on *Mycobacterium leprae* . Am J Trop Med Hyg 21: 446–449. 4559066 10.4269/ajtmh.1972.21.446

[55] Levy L, Shepard CC, Fasal P (1976) The bactericidal effect of rifampicin on *M. leprae* in man: A) Single doses of 600, 900 and 1200 mg; and B) daily doses of 300 mg. Int J Lepr Other Mycobact Dis 44: 183–187. 776856

[56] Ji B, Perani EG, Petinon C, Grosset JH (1992) Bactericidal activities of single or multiple doses of various combinations of new antileprosy drugs and/or rifampin against *M. leprae* in mice. Int J Lepr Other Mycobact Dis 60: 556–561. 1338596

[57] Ji B, Sow S, Perani E, Lienhardt C, Diderot V, et al (1998) Bactericidal activity of a single-dose combination of ofloxacin plus minocycline, with or without rifampin, against *Mycobacterium leprae* in mice and in lepromatous patients. Antimicrob Agents Chemother 42: 1115–1120. 9593137 10.1128/aac.42.5.1115 PMC105755

[58] Ji B, Grosset J (2000) Combination of rifapentine-moxifloxacin-minocycline (PMM) for the treatment of leprosy. Lepr Rev 71 Suppl: S81–87. 11201894 10.5935/0305-7518.20000074

[59] Hastings RC, Chehl SK (1991) Chemotherapy of leprosy in multibacillary nude mice. Indian J Lepr 63: 350–355. 1804889

[60] Banerjee DK, McDermott-Lancaster RD (1992) An experimental study to evaluate the bactericidal activity of ofloxacin against an established *Mycobacterium leprae* infection. Int J Lepr Other Mycobact Dis 60: 410–415. 1474279

[61] Tomioka H, Saito H (1993) Therapeutic efficacy of some new quinolones and a combination of ofloxacin with rifampin against *Mycobacterium leprae* infection induced in athymic nude mice. Int J Lepr Other Mycobact Dis 61: 250–254. 8396614

[62] Ji B, Perani EG, Petinom C, Grosset JH (1996) Bactericidal activities of combinations of new drugs against *Mycobacterium leprae* in nude mice. Antimicrob Agents Chemother 40: 393–399. 8834886 10.1128/aac.40.2.393 PMC163122

[63] Banerjee DK, McDermott-Lancaster RD, McKenzie S (1997) Experimental evaluation of possible new short-term drug regimens for treatment of multibacillary leprosy. Antimicrob Agents Chemother 41: 326–330. 9021187 10.1128/aac.41.2.326 PMC163709

[64] Moet FJ, Pahan D, Oskam L, Richardus JH (2008) Effectiveness of single dose rifampicin in preventing leprosy in close contacts of patients with newly diagnosed leprosy: Cluster randomised controlled trial. BMJ 336: 761–764. 18332051 10.1136/bmj.39500.885752.BE PMC2287265

[65] Feenstra SG, Pahan D, Moet FJ, Oskam L, Richardus JH (2012) Patient-related factors predicting the effectiveness of rifampicin chemoprophylaxis in contacts: 6 year follow up of the COLEP cohort in Bangladesh. Lepr Rev 83: 292–304. 23356030

[66] Ji B, Chauffour A, Andries K, Jarlier V (2006) Bactericidal activities of R207910 and other newer antimicrobial agents against *Mycobacterium leprae* in mice. Antimicrob Agents Chemother 50: 1558–1560. 16569884 10.1128/AAC.50.4.1558-1560.2006 PMC1426933

[67] Burgos J, de la Cruz E, Paredes R, Andaya CR, Gelber RH (2011) The activity of several newer antimicrobials against logarithmically multiplying *M. leprae* in mice. Lepr Rev 82: 253–258. 22125933

[68] Rosenthal IM, Tasneen R, Peloquin CA, Zhang M, Almeida D, et al (2012) Dose-ranging comparison of rifampin and rifapentine in two pathologically distinct murine models of tuberculosis. Antimicrob Agents Chemother 56: 4331–4340. 22664964 10.1128/AAC.00912-12 PMC3421552

[69] Almeida DV, Converse PJ, Li SY, Tyagi S, Nuermberger EL, et al (2013) Bactericidal activity does not predict sterilizing activity: The case of rifapentine in the murine model of *Mycobacterium ulcerans* disease. PLoS Negl Trop Dis 7: E2085. 23469308 10.1371/journal.pntd.0002085 PMC3585034

